# A Dielectric-Filled Waveguide Antenna Element for 3D Imaging Radar in High Temperature and Excessive Dust Conditions

**DOI:** 10.3390/s16081339

**Published:** 2016-08-22

**Authors:** Ding Xu, Zhiping Li, Xianzhong Chen, Zhengpeng Wang, Jianhua Wu

**Affiliations:** 1 School of Electronic and Information Engineering, Beihang University, Beijing 100191, China; lzp@buaa.edu.cn (Z.L.); bee1030@163.com (Z.W.); wjh@buaa.edu.cn (J.W.); 2 School of Automation & Electrical Engineering, University of Science and Technology Beijing, Beijing 100083, China; cxz@ustb.edu.cn

**Keywords:** 3D imaging, dielectric-filled antenna, microstrip-to-waveguide transition, industrial radar

## Abstract

Three-dimensional information of the burden surface in high temperature and excessive dust industrial conditions has been previously hard to obtain. This paper presents a novel microstrip-fed dielectric-filled waveguide antenna element which is resistant to dust and high temperatures. A novel microstrip-to-dielectric-loaded waveguide transition was developed. A cylinder and cuboid composite structure was employed at the terminal of the antenna element, which improved the return loss performance and reduced the size. The proposed antenna element was easily integrated into a T-shape multiple-input multiple-output (MIMO) imaging radar system and tested in both the laboratory environment and real blast furnace environment. The measurement results show that the proposed antenna element works very well in industrial 3D imaging radar.

## 1. Introduction

Three-dimensional (3D) microwave imaging technology has made great development in the last 20 years. Because of the advantage of strong penetrability and strong anti-jamming ability, compared to optical and ultrasonic imaging, the microwave 3D imaging has been widely used in synthetic aperture radar (SAR), human body security scanners, breast cancer detection, and through-wall and ground-penetrating radar [1,2,3,4]. 3D imaging detection technology is also required in some industrial areas with harsh environments, high temperatures and excessive dust conditions, for example. The burden surface information is very important for security and high-efficient production like in blast furnace and lime kiln. Different types of detection methods—laser and ultrasonic—are used to obtain the burden surface environment. However, the laser is easily interfered with by dust, and the speed of ultrasonic is easily interfered with by temperature. Stationary single-point microwave-level radar have been successful applied in these fields [5,6]. Considering the microwave wavelength is longer and the wave velocity is not sensitive to temperature, microwave imaging technology has become a promising technology for these areas.

Microwave imaging requires either a real aperture or a synthetic aperture to obtain cross-range resolution, such as phased array radar or SAR. Multiple-input multiple-output (MIMO) radar is a new radar type and has attracted more and more attention recently [7,8]. It uses multiple, spatially distributed transmitters and receivers to obtain the number of virtual observation channels far more than the actual number of antenna channels. It improves the performance of the radar imaging with sparse array, which is suitable for high-temperature application environments. The sparse array has extra space for an additional protection device, which increases the reliability of the system compared to the dense array.

Radome is normally used for antenna protection in bad operating environments [9]. A radar array system [10] for burden surface imaging operates around 77 GHz with the microstrip antenna elements also needed for a fire brick radome. But under these excessive dust with high temperature conditions, the dust will soon agglomerate and adhere to the radome, and then the microwave signal cannot penetrate out. One of the feasible solution is to design a type of antenna element that can operate in the harsh environment directly. Some types of antenna elements—such as leaking wave antenna [11] and open-ended waveguide [12]—are often used in array design. The leaky wave antenna is a traveling wave antenna and often used for high directional beam and low sidelobe applications. Because of the traveling wave, the leaky wave antenna is sensitive to frequency, and the beam pattern will scan with frequency, so the leaky wave antenna is often used in narrowband application. Secondly, the radiation element of the leaky wave antenna often cannot control independently, so the leaky wave antenna is mainly used as an antenna element with fan beam in one-dimensional linear arrays such as automotive collision avoidance radar or monopulse antenna arrays. The aperture of the open-ended waveguide antenna and horn antenna cannot be smaller than 0.5λ in both of the two mutually perpendicular directions because of the cutoff wavelength in the metal waveguide. The array composed of open-ended waveguide antenna usually has a limited scan angle in order to reduce the grating lobe. The Vivaldi microstrip antenna is widely used in wideband array, but this kind of antenna is difficult to use in high temperatures and dust environments. The dielectric rod antenna was reported as being used in a millimeter imaging system recently [13], but the antenna beam is narrow and cannot be applied to large-angle imaging.

In this paper, a microstrip-fed dielectric-filled waveguide antenna element is proposed for the industrial imaging radar which can operate in harsh environments. Firstly, the working frequency of the antenna was chosen by investigating the relationship of the reflection coefficient of the tested material with frequency. Then, the antenna element was designed through the design of three parts. Thirdly, a prototype of the proposed antenna element was designed and implemented to prove the concept. In the end, we used the proposed antenna element to design and manufacture a T-shape MIMO imaging radar, and the test results proved that the proposed antenna element can be a promising solution for industrial 3D imaging radar.

## 2. Working Frequency Selection

For an imaging radar, the reflection characteristics of the measured medium affect the strength of the echo signal. Considering the size and the cost of the industrial radar, the reflection characteristics at X- and Ku-bands were investigated. The raw materials inside the blast furnace mainly consist of iron ore and coke. The iron ore and coke ratio in volume is about 1:2. The iron ore is a medium with high metal content. The radar cross-section (RCS) characteristics are similar to the rough surface metal and increase with increasing frequency. The coke is a porous carbon medium and the reflection characteristics was studied by experiment.

**Figure 1:**
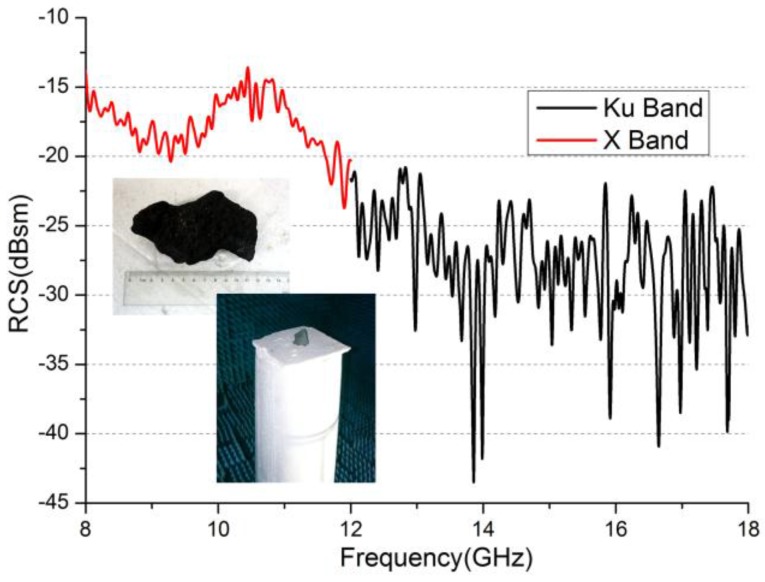
The radar cross-section (RCS) test result of the coke sample.

We measured the RCS of a typically sized coke at 8–18 GHz in compact antenna test range (CATR) in Beihang University (Beijing, China). The dimensions of the coke being tested were about 120 mm × 80 mm × 70 mm. The measurement result is shown in Figure 1. From the result, it was determined that the porous carbon has an absorption effect on microwaves, and the reflection decreases with increasing frequency. The average RCS at X-band is about 10 dBsm higher than it is at Ku-band as can be seen from the Table 1. Based on this measurement, X-band was selected as the working frequency band of the imaging radar, and the antenna element was designed at this frequency band.

## 3. Antenna Element Design Concept

A simple T-shape MIMO antenna array was applied in our industrial 3D imaging radar. In order to eliminate grating lobe of the synthesis virtual array, the distance between two antenna elements should less than 0.5λ. In this specific application, the imaging range mainly focused on the ±30 degrees of the scope of conical whose axis was vertical to the antenna array aperture. The element spacing was set to 0.5λ to obtain a bigger aperture without grating lobes, as the imaging angle range was less than ±41 degrees. As the working frequency was X-band, the aperture of the antenna element was set to 18 mm.

**Figure 2:**
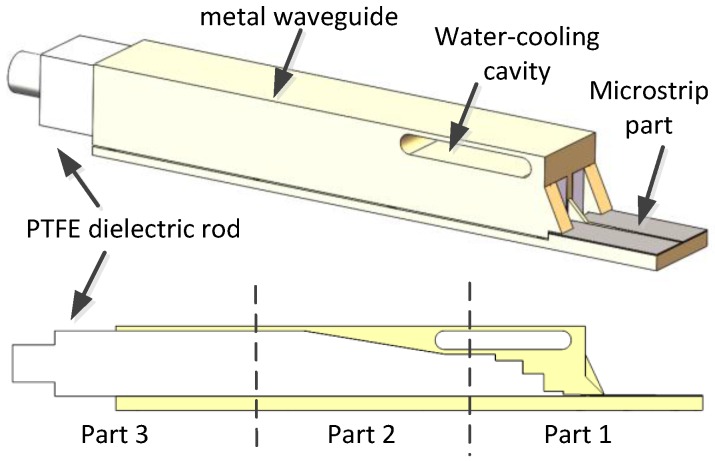
Structure of the proposed antenna element.

The geometry of the proposed antenna element is shown in Figure 2. It is a microstrip-fed dielectric-filled waveguide antenna element. The microstrip direct feed circuit saves microwave connectors and precisely controls amplitude and phase at a low cost. The Polytetrafluoroethylene (PTFE) dielectric rod within the metal waveguide reduces the cutoff frequency and isolates the feed circuit from the outside dust and high temperature environment which performs sealing function. A water-cooling cavity is embedded in the antenna element to enhance the cooling ability and further protect the feed circuit.

The antenna element design is simply broken down into three parts. Part 1 is a microstrip-to-dielectric-filled-rectangle-waveguide transition, part 2 is rectangle-waveguide-to-square-waveguide transition, and part 3 is the dielectric rod extended out of the waveguide for radiation and matching purpose. The CST MICROWAVE STUDIO^TM^ (Darmstadt, Germany) was used for full wave electromagnetic simulation and optimization.

### 3.1. Part 1 Design

**Figure 3:**
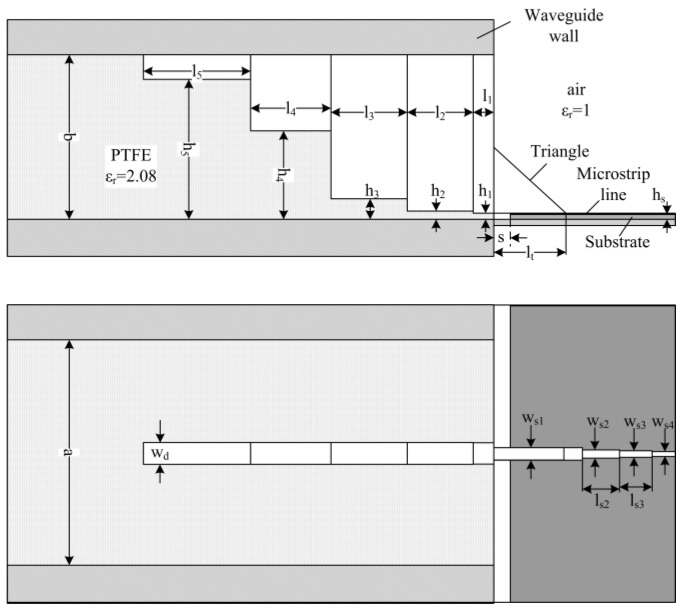
Detailed structure of the microstrip-to-dielectric-filled-waveguide transition.

Many studies on microstrip-to-waveguide transition are reported in literatures [14,15,16]. A single ridged waveguide transition model was designed in our application. The detailed geometry of the transition is shown in Figure 3. The transition contains a microstrip-to-dielectric-filled-ridged-waveguide transition and a ridged-waveguide-to-dielectric-filled-waveguide transition.

As discussed before, the section size of the rectangle waveguide was 18 mm. Considering the thickness of the metal waveguide wall, which was about 2 mm, the dimension of the wide side of the waveguide (a) was set to 14 mm. The cutoff frequency of the TE10 mode in the rectangular waveguide filled with PTFE was 7.415 GHz. The narrow side of the waveguide (b) was set to 9 mm. So the characteristic impedance of the waveguide was 500 Ω at 10 GHz.

Rogers 4350B with a dielectric constant of 3.66 and a thickness of 0.254 mm was used as the substrate for the microstrip line. The width of 50 Ω microstrip line on this substrate was 0.54 mm. If we used the 50 Ω microstrip line to feed the ridged waveguide directly, the width of the triangle metal block should be set to 0.54 mm, the same width of the microstrip line. The size was a bit too small for ordinary precision metal machining, so we chose the width of the triangle transition section to be 1 mm to reduce processing difficulty and enlarge dimensional tolerance, which reduces machining cost. The characteristic impedance for the microstrip line with width of 1 mm is 33.8 Ω. A 2-order Chebyshev quarter-wave impedance-matching unit was designed to match these two sections. The optimized dimensions—w_s2_, l_s2_, w_s3_, l_s3_—are shown in Table 2.

**Figure 4:**
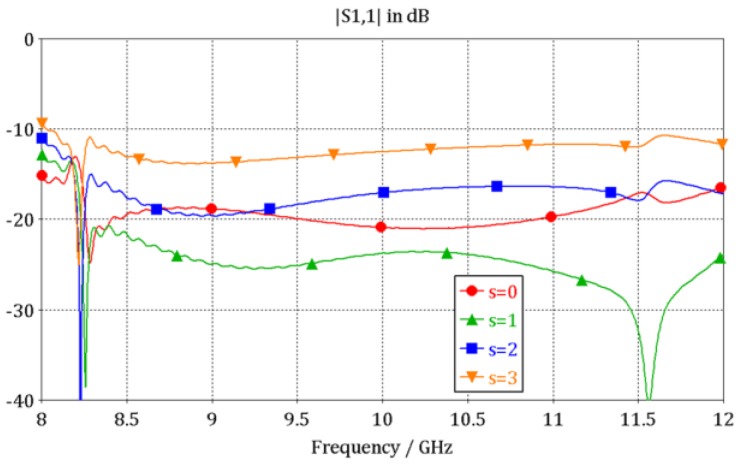
The return loss of part 1 with different lengths (s).

A triangle metal block attached to the ridge was used to connect the microstrip line to the ridge waveguide. The triangle block was an isosceles right triangle and the size of the two right angle side was l_t_. In the triangle transition section, the metal block with the microstrip line together are considered as the thickened conductor microstrip line. As the metal segment height increases, the equivalent microstrip line becomes thicker, which leads to increased capacitive characteristic. A segment of air medium is introduced which provides an inductive characteristic in order to get good reflection performance at working frequency band. The length (s) of the segment was studied through simulation and the result is shown in Figure 4. It can be observed that the optimized air medium segment improves the reflection characteristics and the optimized length (s) is 1 mm when the triangle metal right angle side length (l_t_) is 4 mm.

A standard 4-order Chebyshev transformer based on λ_g_/4 sections of different heights were employed for the dielectric-filled-ridged-waveguide-to-dielectric-filled-waveguide transition. Considering the difficulty of processing, the width of the ridge (wd) was set to 2 mm. The power–voltage definition of impedance of the ridged waveguide was used to design the impedance-matching structure [17], and the optimized dimensions of the transformer (l_2_, h_2_, l_3_, h_3_, l_4_, h_4_, l_5_, h_5_) are also listed in Table 2. The final reflection characteristics of part 1 are shown in Figure 4, which are indicated by the green curve that s = 1, and the return loss is less than −20 dB from 8.2 GHz to 12 GHz.

### 3.2. Part 2 Design

**Figure 5:**
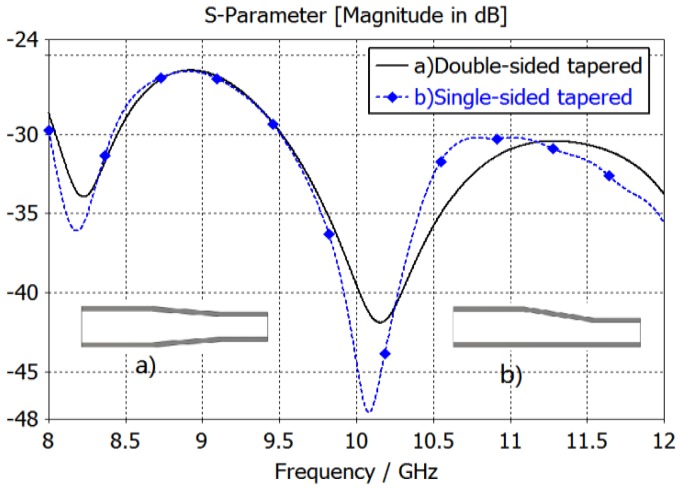
Return loss of different type transformers.

Square cross-section of the waveguide was applied in order to achieve substantially equal E-plane and H-plane radiation patterns. A transformer was required to suppress reflection between the rectangular waveguide and square waveguide. Two different tapered waveguide transformers were used for the rectangle-waveguide-to-square-waveguide. The simulation results are shown in Figure 5. The results from the two types of transformers are quite similar. Considering the single side tapered transformer has more space for a water-cooling cavity, it was employed in our final antenna element design.

### 3.3. Design of the Radiation Section

**Figure 6:**
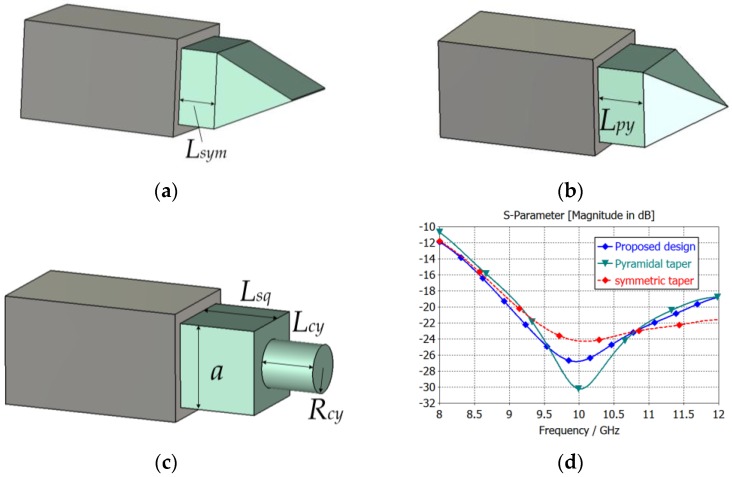
(**a**) Symmetric taper dielectric rod; (**b**) pyramidal taper dielectric rod; (**c**) proposed design; (**d**) reflection characteristics of the different radiation segments.

Tapering dielectric rod antenna is often used as a dielectric waveguide antenna radiation section, such as symmetric tapering or pyramidal tapering section [18,19], as shown in Figure 6a,b. At high temperatures and in harsh environments, in order to increase the reliability of the antenna, the dielectric rod length extending out of the metal waveguide should be as short as possible. Meanwhile, the 3 dB beam width of the antenna can cover the test area, and was ±35° in this test scenario. A two-stage dielectric rod antenna consists of a square dielectric rod and a matching cylindrical segment is proposed as the radiation section of the antenna element. The length of the square dielectric section extending from the waveguide is L_sq_, the length of the cylindrical segment is L_cy_, and the cylinder radius is R_cy_. Cylindrical axis is coaxial with the axis of the square cylinder, as shown in Figure 6c. Because the antenna pattern of dielectric rod antenna depends on the length of the dielectric, all three types of antennas are set to the same dielectric length, 22 mm. Each one is optimized to achieve the best return loss at X-band. The parameters optimized by the simulation are shown in Table 3. The return loss curves of part 3 are shown in Figure 6d. Compared with the tapering rod antennas with the same antenna pattern, the symmetric tapering antenna provides the widest band whose return loss is below −20 dB, but the average return loss is bigger. The pyramidal tapering antenna has the best match at the center frequency, but the working frequency band is narrowest. The proposed dielectric waveguide antenna has a low return loss below −20 dB from 9 GHz to 11.5 GHz, and at the same time, lacking a pointy tip makes the proposed antenna more robust, and lacking an inclined plane makes the antenna easy to process. So the proposed antenna is more suitable for use in harsh industrial conditions.

## 4. Prototype Measurement

**Figure 7:**
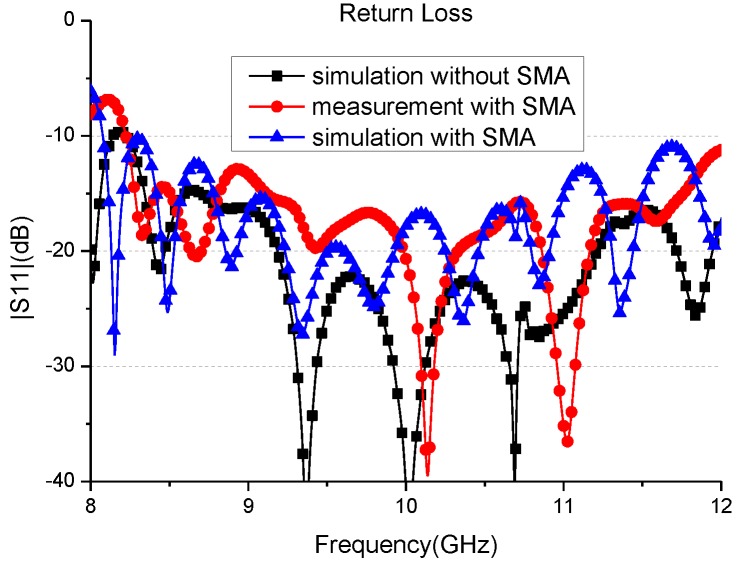
The reflection characteristics of the proposed antenna element.

**Figure 8:**
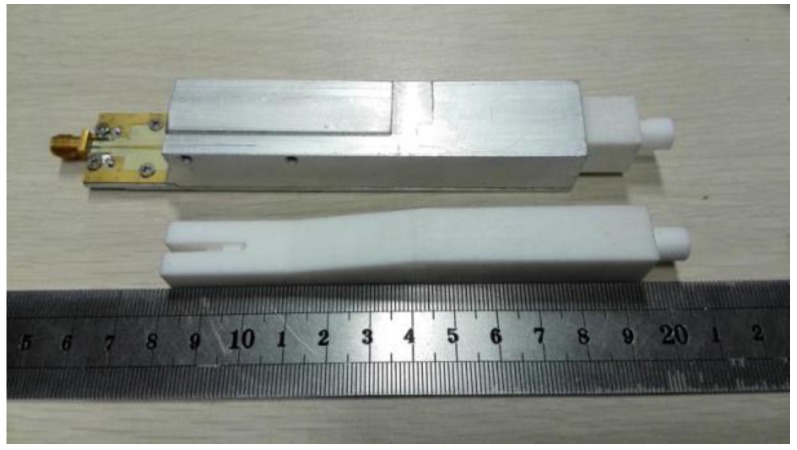
Photograph of a prototype of the proposed antenna element.

**Figure 9:**
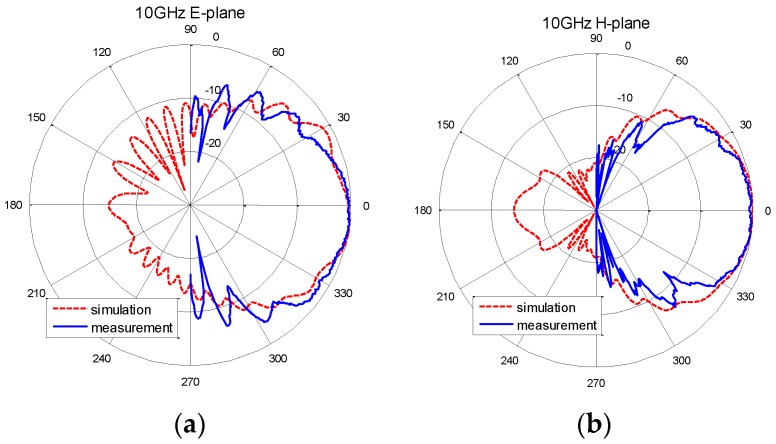
(**a**) The E-plane pattern and (**b**) H-plane pattern of the proposed antenna element at 10 GHz.

The whole antenna element was simulated and the reflection characteristics are shown in Figure 7. The return loss of the whole antenna is below −20 dB from 9.2 GHz to 11.2 GHz. A prototype of the antenna based on the concept was fabricated as shown in Figure 8. The antenna element has been measured in CATR of Electromagnetic Engineering Laboratory, Beihang University. The reflection characteristics are shown in Figure 7, and the antenna patterns at 10 GHz in E-plane and H-plane are shown in Figure 9, where the simulation results are also shown. It can be observed that the measured S11 is bigger than the simulated result due to the introduction of additional Voltage Standing Wave Ratio (VSWR)of SMA (SubMiniature version A)-to-microstrip transition, the machining error, and assembly error. The measured return loss is less than −15 dB from 9.2 GHz to 11.2 GHz with the SMA. The antenna pattern test results show that the 3 dB beamwidth in E-plane is 60° and in H-plane it is 64°. The antenna element radiation pattern perfectly covers the test angle range of ±30°.

## 5. Array Arrangement Method

**Figure 10:**
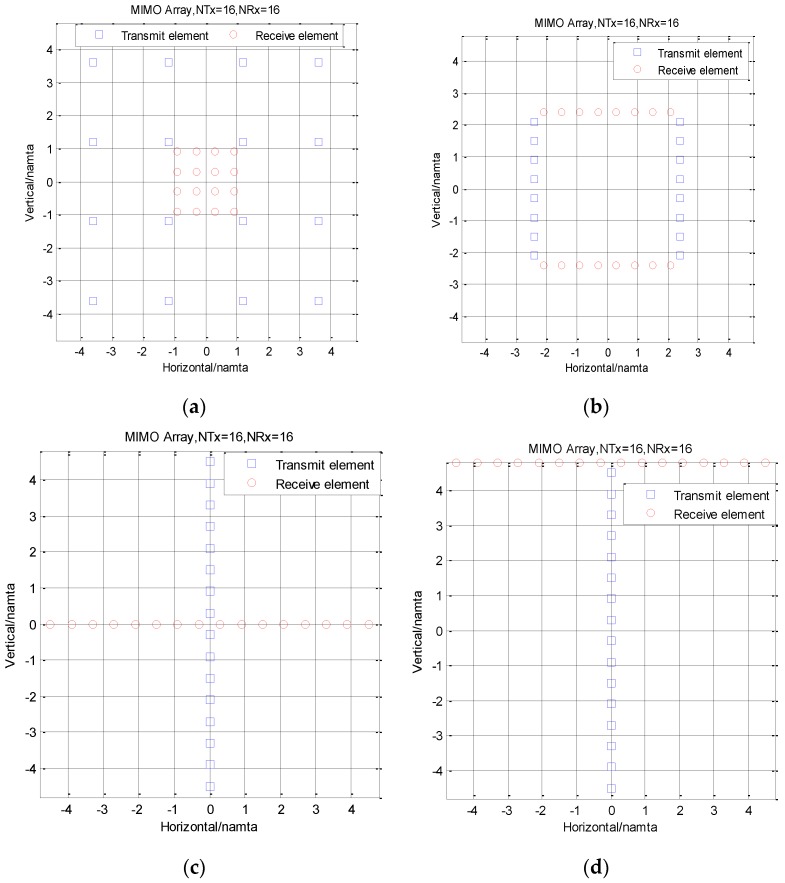
Different types of multiple-input multiple-output (MIMO) array arrangements: (**a**) square type I array; (**b**) square type II array; (**c**) cross type array; (**d**) T type array.

There are many kinds of array arrangement methods that can obtain a 16 × 16 equivalent virtual array. Four kinds of array with receiving element spacing of 0.6λ are compared here. The four kinds of arrays are square type I array, square type II array, cross type array, and T type array, as shown in Figure 10. The corresponding array characteristics are shown in Table 4; the array size was calculated with antenna element size of 0.6λ. The four arrays can obtain the same equivalent virtual array. The square type I array has the complex feed network and the cross type array has the two arrays cross at the central point. The square type II array has the smallest actual array size and has great potential to be used in our future systems. The T type array has the simplest feed network and is capable of using planar array feed technology. In addition, the vertical distance between transmitting array and receiving array in T type array is does not need to be strictly limited as in the other three arrays, so its advantage is that the distance can be increased appropriately to reduce the mutual coupling between the transmitting array and the receiving array. Therefore, the T type array was selected in this application.

## 6. MIMO Imaging Radar Fabrication and Testing

**Figure 11:**
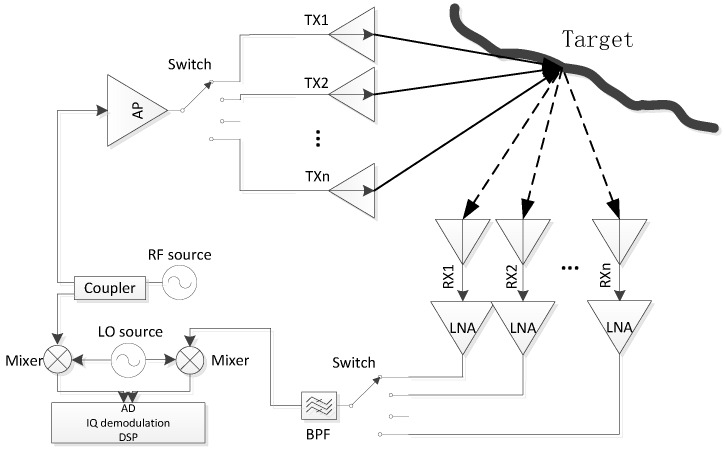
The system block diagram of the imaging radar.

The system block diagram of the imaging radar is shown in Figure 11. The system contains 16 transmit antennas and 16 receive antennas, two 1:16 switch matrices, and one microwave receiver. The transmit antennas and receive antennas form switched arrays which are switched through microwave switches. The wideband microwave signal transmitted by one transmit antenna is reflected by the target, and the echo signal is received by the 16 receive antennas in sequence. Then another signal emitted by another transmit antenna is also received by receive antennas. So, the 16 transmitting channels and 16 receiving channels form 256 signal channels. The microwave receiver is a superheterodyne receiver and the IQ(In-phase and Quadrature) signals after demodulation are used in image processing by a digital signal processing (DSP) system.

Fifteen SPDT (single pole double throw) switch chips form a 1:16 switch matrix, and the switch chip is HMC547LC3 from Hittite corporation. The measured insert loss of the switch array is about 10 dB at 10 GHz. The power of transmit signal is about 10 dBmw and the noise floor of the receiver is −100 dBm@10 GHz. The gain of LNA(Low Noise Amplifier) is 25 dB and the synthesis gain of receiver array is 12 dB. According to the radar Equation (1), for the target of about 8 meters from the antenna, the radar can detect a target of RCS below −40 dBsm. The main technical specifications of the radar system are shown in Table 5.

(1) $$P_{t}=\frac{P_{si(\min)}{\left(4\pi \right)}^{3}R_{\max}^{4}FL_{1}L_{2}L_{3}}{G_{t}^{2}\lambda^{2}\sigma_{\min}}$$

**Figure 12:**
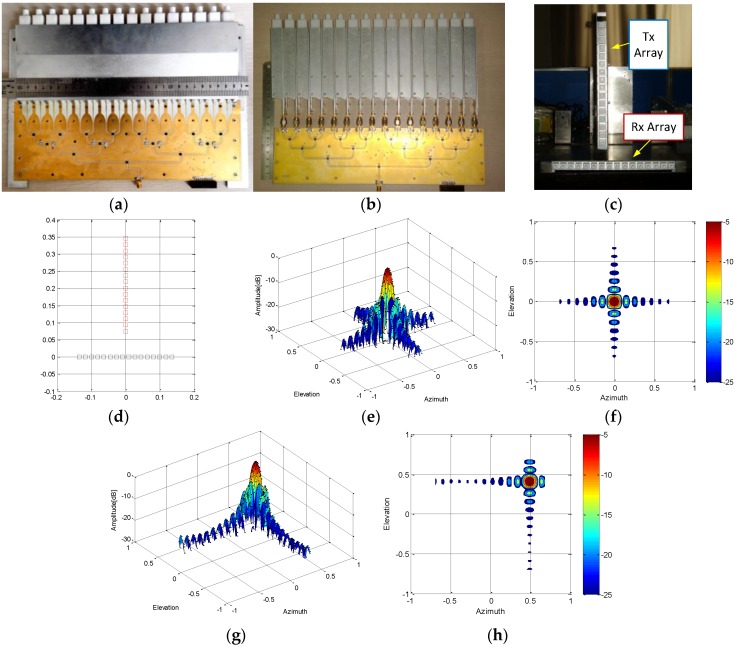
(**a**) Receiving array; (**b**) transmitting array; (**c**) T-shape MIMO radar; (**d**) array topology; (**e**) beam pattern focus on center position; (**f**) contour plot of beam pattern at center position; (**g**) beam pattern focus on (40°,40°); (**h**) contour plot of beam pattern at edge position.

The proposed antenna elements integrated into a T-shape MIMO radar system. The transmitting and receiving arrays, each containing 16 antenna elements, are both linear arrays and perpendicular to each other, as shown in Figure 12a–c. The receiving array is directly fed by the microstrip. Additional SMA adapters were introduced in the transmitting array in order to provide the same polarization as the receiving array. Based on the MIMO radar imaging algorithm [20], the T-shape MIMO array is the equivalent of 256 antenna elements array. The point spread function (PSF) of the array is obtained with numerical simulation and the simulation results are shown in Figure 12d–h. The angular resolution of the MIMO array is about 3 degrees and the max sidelobe level is about −13 dBc.

**Figure 13:**
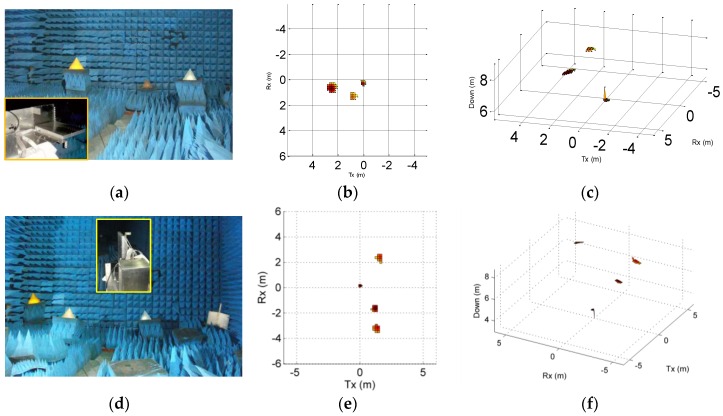
Test scenarios and 3D imaging results (**a**) Tx array placed in horizontal direction with three reflectors; (**b**) 2D front view obtained by MIMO radar; (**c**) 3D view obtained by MIMO radar; (**d**) Rx array placed in horizontal direction with four reflectors; (**e**) 2D front view obtained by MIMO radar; (**f**) 3D view obtained by MIMO radar.

The MIMO radar was tested in an anechoic chamber to verify 3D imaging capability. Corner reflectors as the test targets were placed at 4–9 m away from the radar aperture and the cross space between them was about 1–2 m. The corner reflectors were arranged in the horizontal direction with different heights. It is difficult to arrange the targets over a large area in height direction in the laboratory, so we flipped the radar to test imaging capability in both directions. Firstly, the Tx array was placed in the horizontal direction and tested, as shown in Figure 13a. Then, it was turned 90° from the radar to put the Rx array in the horizontal direction and another test performed. The imaging capability of the radar system in both horizontal and vertical directions was tested based on this method. The working frequency of the MIMO radar was from 9.2 GHz to 11.2 GHz, and the number of the frequency points was 1024.

Arrangement of test scenarios and test results are shown in Figure 13. It can be seen that the radar can image in wide angles in both directions, and the imaging results coincide with the target arrangement position. The result shows that the T-shape MIMO radar built with the proposed antenna elements can be used for 3D measurement and high accuracy images are achieved.

**Figure 14:**
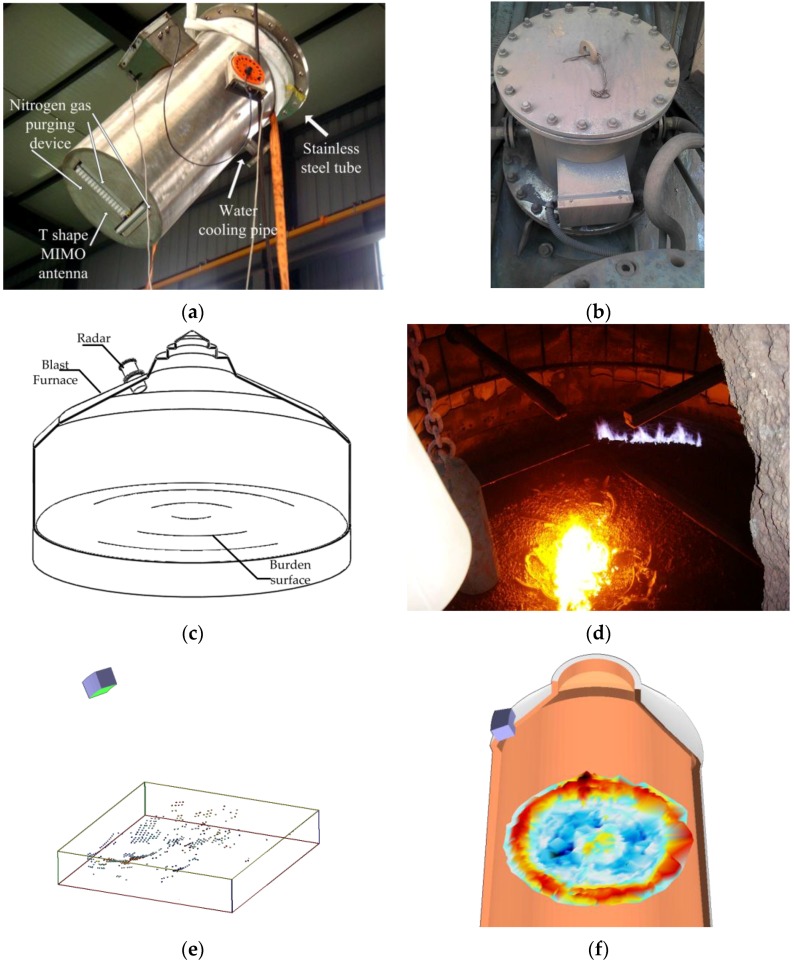
(**a**) MIMO radar with stainless steel protective tube; (**b**) the radar mounted on a real blast furnace; (**c**) the schematic diagram of radar installing; (**d**) the real burden surface in the blast furnace; (**e**) the 3D point cloud obtained by the radar; (**f**) reconstructed burden surface.

The T-shape MIMO radar with proposed antenna elements was housed in a stainless steel protective tube and mounted on blast furnace #7 of Wuhan Iron and Steel Group Corp., as shown in Figure 14a,b. The protective tube has a water-cooling pipe and a nitrogen purging device. Several temperature sensors were installed close to the antenna aperture in the tube to monitor the temperature. With these protection measures, the temperature of the MIMO radar inside the tube was kept below 60 °C, even when the external temperature exceeded 200 °C.

The industrial 3D imaging radar mounted on the blast furnace had an angle of inclination so the radar aimed at the center of the burden surface, as shown in Figure 14c. Based on MIMO radar imaging data and blast furnace internal dimensions, prior information was obtained that supports appropriate spatial filtering technology to remove interference signals. It was expected that a more accurate 3D burden surface image could be obtained. The discrete point cloud data obtained by the radar, filtered by amplitude and range, is shown in Figure 14e; the threshold for amplitude filtering is −20 dB and the range of vertical height is ±1.5 m. It can be observed that the part far from the radar has a less effective point because of the large oblique incidence angle and longer distance. We used the circular symmetry and extrapolation method based on burden surface distribution model to estimate this part of the data. In the future, we should study the novel MIMO array that has narrow mainlobe with low sidelobe. A reconstructed blast furnace burden surface is shown in Figure 12f. A color scale was used to distinguish the different heights, where red to blue represents high to low.

## 7. Conclusions

A novel dielectric-filled waveguide antenna element for 3D burden surface imaging industrial radar is proposed in this paper. The relationship between frequency and the reflection characteristics of the main material has been studied and the working frequency of the antenna has been chosen. The metal waveguide shell with water-cooling cavity and the filled dielectric can separate the feed circuits from high temperatures and dust. An air gap is used to improve the impedance matching for microstrip-to-ridged-waveguide transition. A novel dielectric radiation segment—including a square column and a cylinder dielectric rod—extending out of the waveguide was developed. The geometry provides better return loss performance and higher reliability compared to traditional tapering dielectric rod antennas. According to the simulated result, a prototype of the antenna element working at X-band was fabricated and tested. Good accordance is obtained between simulated and measured results. A prototype of a T-shape 3D imaging MIMO radar was designed and assembled. The MIMO radar was tested both in the laboratory environment and real blast furnace environment. The measurement results show that the radar can obtain relatively accurate 3D burden surface information in harsh environments.

## Figures and Tables

**Table 1:** Average RCS test results of coke sample.

Frequency (GHz)	8–12 (VV)	12–18 (VV)
Average RCS (dBsm)	−17	−28

**Table 2:** Dimensions (mm) of the microstrip-to-dielectric-filled-waveguide transition.

*s*	*h_1_*	*h_2_*	*h_3_*	*h_4_*	*h_5_*	*a*	*w_d_*	*w_s2_*	*w_s3_*	*w_s4_*
1	0.271	0.4	1.1	4.8	7.6	14	2	0.9	0.68	0.54
*l_t_*	*l_1_*	*l_2_*	*l_3_*	*l_4_*	*l_5_*	*b*	*w_s1_*	*l_s2_*	*l_s3_*	*h_s_*
4	1	3.7	4.13	4.5	5.83	9	1	4.43	4.48	0.254

**Table 3:** Parameters (mm) of the proposed radiation section.

*L_sq_*	*a*	*L_cy_*	*R_cy_*	*L_sym_*	*L_py_*
13	14	9	4	7	8.8

**Table 4:** Comparison of array characteristics.

*Array Type*	*d_Tx_*	*d_Ty_*	*d_Rx_*	*d_Ry_*	*Array Size*
square I	2.4λ	2.4λ	0.6λ	0.6λ	7.8λ × 7.8λ
square II	4.8λ	0.6λ	0.6λ	4.8λ	5.4λ × 5.4λ
cross type	0	0.6λ	0.6λ	0	9.6λ × 9.6λ
T type	0	0.6λ	0.6λ	0	9.6λ × 10.2λ

**Table 5:** Technical specifications of the radar system.

Parameter		Value
Center frequency	f_c_	10 GHz
Bandwidth	B	2 GHz
Number of samples	N	1024
Sweep period	T_t_	15 ms
Virtual receiving array number	M	256
Array aperture	D	288 × 342 mm^2^
Range resolution	d_r_	15 cm
Angular resolution	d_θ_	3 deg
Imaging time for one frame	T_f_	45 s
